# Validity and reliability of the Romanian version of the Hill-Bone compliance to high blood pressure therapy scale

**DOI:** 10.3389/fphar.2024.1256848

**Published:** 2024-03-07

**Authors:** Răzvan-Nicolae Rusu, Radu Sebastian Gavril, Daniela Carmen Ababei, Ioana Macadan, Andrei Ciobîcă, Camelia Nicolae, Răducu Ionuț Popescu, Walther Bild, Veronica Bild

**Affiliations:** ^1^ Pharmacodynamics and Clinical Pharmacy Department, Faculty of Pharmacy, “Grigore T. Popa” University of Medicine and Pharmacy, Iasi, Romania; ^2^ Medical I Department, Faculty of Medicine, “Grigore T. Popa” University of Medicine and Pharmacy, Iasi, Romania; ^3^ Physiology Department, Faculty of Medicine, “Grigore T. Popa” University of Medicine and Pharmacy, Iasi, Romania; ^4^ Department of Internal Medicine and Cardiology, “Carol Davila” University of Medicine and Pharmacy, “Prof. Dr. Th. Burghelia” Hospital, Buchares, Romania; ^5^ Center of Biomedical Research of the Romanian Academy, Iasi, Romania

**Keywords:** adherence, hypertension, questionnaire, Hill-Bone, validity, reliability, Romanian

## Abstract

Romania is considered a country with high cardiovascular risk, arterial hypertension and its complications accounting for about 60% of total deaths. The management of high blood pressure often involves a combination of both therapeutic regimens as well as lifestyle changes, to which patients have to be adherent. In order to assess patients adherence to professionals’ recommendations, validated tools are needed. The aim of our study was to translate, culturally adapt and validate the Hill-Bone Compliance to High Blood Pressure Therapy Scale into Romanian. The study included 215 participants from Iasi, North-Eastern Romania. The internal consistency of the instrument was measured with Cronbach’s alpha coefficient, while the construct validity was determined using exploratory factor analysis and principal component extraction with promax rotation. Sampling adequacy and appropriateness of data for factor analysis was measured using Kaiser-Meyer-Olkin (KMO) statistics and Bartlett’s test of sphericity. Our statistical analysis revealed a Cronbach’s alpha coefficient of 0.733 (73.3%) and a Kaiser-Meyer-Olkin (KMO) Measure of Sampling Adequacy of 0.697. The chi square test demonstrated that the overall perfect adherence was not significantly associated with the number of medications taken per day variable (*p* = 0.721). The Romanian version of the Hill-Bone Compliance to High Blood Pressure Therapy Scale demonstrated suitability for its use in evaluating adherence in the intended population.

## 1 Introduction

Cardiovascular diseases (CVDs) are the most well represented causes of morbidity and mortality worldwide. The major risk factor for CVDs, regardless of sex or ethnicity is hypertension (HT). It is indirectly the leading cause of death as well as among the top causes of disability globally (Wierzejska et al., 2020), being involved in the development of heart failure, myocardial infarction, stroke and renal failure. It is estimated that one billion adults have hypertension, number which is expected to reach 1.5 billion by the year of 2025 (Iqbal and Jamal, 2023). Hypertension costs account for over 3% of the $3trillion US national healthcare expenditure (Kirkland et al., 2018).

Romania is an Eastern European country with high cardiovascular risk. The prevalence of high blood pressure is approximately 45.1% (Pop et al., 2021), arterial hypertension alongside its complications accounting for approximately 60% of total deaths (Dorobantu et al., 2018).

The management of hypertension is complex and involves lifestyle modifications as well as antihypertensive drug treatment, which are known strategies for reducing blood pressure. The most important lifestyle recommendations refer to weight control, moderation of alcohol intake, physical exercise, dietary sodium restriction, adequate intake of vegetables, fruits, low-fat dairy products. Preventing obesity is a priority, since this lowers the burden of hypertension as well as other conditions (Parati et al., 2023). Antihypertensive drug treatments include several drug classes, such as thiazide-type diuretics, renin-angiotensin-aldosterone system inhibitors, calcium channel blockers, beta blockers (Chobanian, 2017). Although these methods are efficient in lowering blood pressure, adherence is often problematic. Adherence is defined by the W.H.O. as the extent to which a person’s behavior-taking medication, following a diet and executing lifestyle changes corresponds with agreed recommendations from a healthcare provider (World Health, 2003). In medication-taking behavior studies, the term “adherence” and “compliance” are often interchangeable, although compliance implies that patients passively follow professionals’ recommendations, this term being less preferred (Fraser, 2010; Hamrahian et al., 2022). Adherence levels can differ depending on the specific study and the methods of assessment, being considered that non-adherence can be as high as 50% (Poulter et al., 2020). While there is limited information regarding patients’ adherence to physical exercise, dietary changes and keeping up with medical appointments, it seems that only 40%–50% of hypertensive patients report good levels of adherence to prescribed medication (Al-Daken and Eshah, 2017). For implementing interventions which aim to improve adherence, adequate measures of monitoring adherence are needed in the first place. Ideally, these measures would be able to clearly identify if a certain treatment was administered as prescribed by the professional, while also offer insight into possible reasons for non-adherence. While there is no golden standard which can be applied in every clinical context, questionnaires are often used in adherence-assessment studies due to their non-invasiveness, ease of use, low cost as well as their potential to identify specific barriers to adherence (Lam and Fresco, 2015). One of the most known questionnaires used in patients with hypertension is the Hill-Bone Compliance to High Blood Pressure Therapy Scale. Developed by the School of Nursing from the John Hopkins University in 1999, the 14-item instrument is used to assess adherence levels, while also capturing aspects related to salt reduction and keeping up with appointments (Chia et al., 2021), connecting with the three identified behavioral domains of adherence: 1) reducing sodium intake; 2) appointment keeping; 3) medication taking (Kim et al., 2000). There are several translated and validated versions of this scale in different languages, such as Chinese (Pan et al., 2020), Greek (Chatziefstratiou et al., 2019), Polish (Uchmanowicz et al., 2016), Namibian (Nashilongo et al., 2017) as well as Turkish (Karademir et al., 2009), which highlights the fact that many behavioral aspects are measurable across cultures (Culig and Leppée, 2014). The aim of our study is to take the appropriate steps for the translation, adaptation and validation of this instrument so that it can be used by professionals such as pharmacists or physicians in Romania, since this has not been previously done.

## 2 Materials and methods

### 2.1 Questionnaire

The instrument which was used consisted of an initial socio-demographic part (age, sex, marital status, education, occupation), as well as a health-related information part (disease-history, duration of diagnosis, medication use, specific drugs that are used) and the 14-point Hill-Bone Scale. The Hill-Bone Scale was constructed by Kim et al., 2000 using a Likert model. Respondents can choose the item which indicated the frequency with which a certain situation characterizes their state. The scale is composed of 14 items, with four possible answers, ranging from 1 to 4: 4—all the time; 3—most of the time; 2—some of the time; 1—never. Items are assumed to be additive, which leads to total scores ranging from 14 (minimum) to 56 (maximum). The authors that developed the scale do not recommend specific cut-off points (low/moderate/high noncompliance).

The translation, cultural adaptation and validation of the Hill-Bone scale was done according to the methodology proposed by Sousa and Rojjanasrirat (Sousa and Rojjanasrirat, 2011). This consists of 7 steps, which include a forward translation of the instrument by two independent translators, a comparison of the versions obtained by a third reviewer and members of the research team, a blind back-translation by two other translators of the version obtained in the previous step, a comparison of the back-translated versions, a pilot testing of the pre-final version as well as the full psychometric testing of this instrument, The research team asked the authors that developed the Hill-Bone scale for permission in order to use it in this study. After receiving permission from the Hill-Bone Scales Team, the instrument was translated by two independent translators, one of which had a medical background. Two versions of the instrument resulted, which were presented to a panel of experts which consisted of a pharmacist, a physician and a psychologist. After the translations were verified regarding the meaning, clarity as well as phrasing of the items, a draft-version of the scale was developed with the aid of a third translator. This version was back-translated into English by two other independent translators and compared to the original instrument, for verifying its conceptual, semantic and content equivalence. After discrepancies were solved, an initial Romanian version of the instrument was obtained. This version was then piloted on a group of 57 hypertensive patients, which did not report difficulties in understanding and answering the questions of the instrument. This resulted in the final Romanian version of the Hill-Bone Compliance to High Blood Pressure Therapy Scale, which was then tested for its psychometric properties.

### 2.2 Patients

The study was carried out at a family physician’s office in Iasi, North-Eastern Romania, in 2022 and included 215 hypertensive patients. Data was collected by two pharmacists from the research team (R.N.R. and D.C.A.) which were familiarized with the tool as well as the study protocol. Convenience sampling of hypertensive patients was used to recruit participants from the family pshysician’s office. The purpose of the study was explained to the participants and after their eligibility was verified, patients were invited to participate. Written as well as verbal informed consent were taken from respondents before the interview. Patients were assured regarding their anonymity, the confidentiality of their information as well as the fact that participation is voluntary and they could withdraw from the study whenever they desired. The interview lasted for approximately 15 min and the patients participated during their regular visits to the physician’s office. Patients which were at least 18 years old, who agreed to attend the study, which were diagnosed with hypertension and were undergoing antihypertensive drug therapy were included. Patients under 18 years old, or with cognitive impairment which could have interfered with participating in the interview were not enrolled. The sample size was determined by analyzing the scientific literature and other studies with similar objectives, in accordance with the validation method which was mentioned above. For the psychometric testing of the Polish version of the instrument, Uchmanowicz et al., 2016 reported a sample size of 70 patients, an equivalent of five times the number of variables analyzed while Pan et al. reported that for the Chinese version, a subject-item ratio of 15 was used (Pan et al., 2020). According to the validation methodology used in this study, proposed by Sousa and Rojjanasrirat, it is recommended that at least 10 subjects per item of the instrument scale are used. Thus, a minimum sample of 140 patients is required.

### 2.3 Statistical analysis

Statistical analysis was performed using the Statistical Package for Social Sciences (SPSS) version 28.

Socio-demographic data was analyzed using descriptive statistics (mean ± standard deviation, frequency and percentage). Internal consistency and reliability of the items of the scale was measured using Cronbach’s alpha coefficient. A coefficient value greater than 0.70 is considered to indicate a good internal consistency.

Furthermore, we measured sampling adequacy and appropriateness of data for factor analysis using Kaiser-Meyer-Olkin (KMO) statistics and Bartlett’s test of sphericity. A KMO greater than 0.5 is generally considered acceptable. Construct validity was determined using exploratory factor analysis and principal component extraction with promax rotation. Chi-square test was used to measure if the perfect medication adherence and number of medicines per day variables were related or independent.

## 3 Results

### 3.1 Population characteristics

Our sample consisted of 215 respondents, 67.9% females and 32.1% males. The mean age was 67.49 years old. The majority of respondents were residing at urban residence (90.7%) and were married (66.5%). In addition, 27.9% of the participants graduated only the primary school, followed by 26% of which had a Bachelor’s degree. However, all the participants knew how to read. Furthermore, 95.3% of the individuals owned a mobile phone, while 71.6% had a smart phone. 63.6% drank coffee regularly, 11.2% were smokers and 5.7% consumed alcohol on a regular basis (Table 1).

**TABLE 1:** Socio-demographic characteristics of participants.

Variable		Frequency	Percentage
**Age (years)**	31–40	3	1.4
	41–50	9	4.2
	51–60	18	8.4
	61–70	105	48.8
	71–80	70	32.6
	>80	10	4.7
**Sex**	Male	69	32.1
	Female	146	67.9
**Residence**	Urban	195	90.7
	Rural	20	9.3
**Education Status**	No studies	1	0.5
	Primary School	60	27.9
	Middle School	22	10.2
	High School	39	18.1
	Post secondary school	32	14.9
	Bachelor’s degree	56	26
	Master’s degree	2	0.9
	PhD	3	1.4
**Marital Status**	Married	143	66.5
	Unmarried	11	5.1
	Divorced/separated	10	4.7
	Widow/widower	51	23.7
**Employment Status**	Unemployed	11	5.1
	Employed	41	19.1
	Retired	163	75.8
**Mobile Phone**	Yes	205	95.3
	No	10	4.7
**Smart Phone**	Yes	154	71.6
	No	61	28.4
**Coffee Consumption**	Yes	133	63.6
	Occasionally	10	4.8
	No	66	31.6
**Smoking Habit**	Yes	24	11.2
	Occasionally	3	1.4
	No	182	84.7

### 3.2 Hill-Bone compliance to high blood pressure therapy scale scores

The statistical analysis of adherence showed that the mean score of overall adherence was 51.82 points with a standard deviation of 3.98. In addition, mean scores of medications taking adherence, reduced salt intake and appointment keeping were 34.52 (SD = 2.4), 10.02 (SD = 1.64) and 7.28 (SD = 1.11), respectively. The question “How often do you run out of high blood pressure pills when you feel sick?” had the highest percentage of respondents (93.4%) who answered “never”. Furthermore, only 8.3% respondents scored perfectly to all adherence subscales, while 63.2% were perfectly adherent to appointment keeping and 47.4% were perfectly adherent to the medication taking subscale (Table 2).

**TABLE 2:** Participants’ Hill-Bone Compliance to High Blood Pressure Therapy Scale scores.

Subscales	Level of adherence	Frequency	Percentage
**Adherence to medication taking (9 items)**	Perfect adherence (score = 36)	101	47.4
	Non- adherence (score <36)	112	52.6
**Adherence to reduce salt intake (3 items)**	Perfect adherence (score = 12)	38	18.5
	Non- adherence (score <12)	167	81.5
**Adherence to appointment keeping (2 items)**	Perfect adherence (score = 8)	129	63.2
	Non- adherence (score <8)	75	46.8
**Overall treatment adherence (14 items)**	Perfect adherence (score = 56)	17	8.3
	Non- adherence (score <56)	187	91.7

### 3.3 Face validity

Pre-testing feedback was used to attain the face validity of the Romanian Version of the Hill-Bone Compliance to High Blood Pressure Therapy Scale Questionnaire. Specifically, participants were asked to give feedback regarding any difficulty in understanding the questions. No such difficulties were reported.

### 3.4 Content validity

The original Hill-Bone Compliance to High Blood Pressure Therapy Scale tool has been used in many studies and it has been translated into several languages. These studies evaluated the extent to which it measures adherence to treatment and proved that the Hill-Bone compliance to High Blood Pressure Therapy Scale has a good content validity (Karademir et al., 2009; Nogueira-Silva et al., 2016; Uchmanowicz et al., 2016; Chatziefstratiou et al., 2019). Nevertheless, to maintain the content validity of the Romanian version of the Hill-Bone Compliance to High Blood Pressure Therapy Scale, the translation of the tool was analyzed by an expert panel. After they reviewed the translated manuscript, only minor language corrections were needed.

### 3.5 Internal consistency

Our statistical analysis revealed a Cronbach’s Alpha coefficient of 0.733 (73.3%). Only one item (item 6) had a slightly higher alpha value (0.744) compared to the composite value (0.733). However, given that our composite coefficient value was greater than 0.70 and therefore indicated a high level of internal consistency, we decided not to delete the item in order to enhance reliability. In addition, item-to-total correlation ranged from 0.186 to 0.643. All Corrected Item-Total Correlation, Squared Multiple Correlation and Cronbach’s Alpha if Item deleted can be seen in Table 3.

**TABLE 3:** Internal consistency of analysis of the Romanian version of the Hill-Bone questionnaires.

Items	Corrected item-total correlation	Squared multiple correlation	Cronbach’s alpha if item deleted
Item 1	0.305	0.310	0.722
Item 2	0.414	0.332	0.710
Item 3	0.289	0.205	0.732
Item 4	0.418	0.305	0.709
Item 5	0.383	0.215	0.714
Item 6	0.263	0.152	0.744
Item 7	0.234	0.510	0.728
Item 8	0.359	0.614	0.718
Item 9	0.433	0.396	0.718
Item 10	0.543	0.422	0.702
Item 11	0.643	0.561	0.690
Item 12	0.314	0.316	0.722
Item 13	0.186	0.380	0.732
Item 14	0.415	0.510	0.710

### 3.6 Construct validity

Our statistical analysis indicated that the sample size was adequate for factor analysis with a Kaiser-Meyer-Olkin (KMO) Measure of Sampling Adequacy of 0.697 (a KMO greater than 0.5 is generally considered acceptable). The *p*-value of the Bartlett test of homogeneity of variances (sphericity) was lower than 0.001, which demonstrates that the variance was different for various components from 1 to 14 items of questionnaires. Furthermore, the significant Bartlett test result (*p* < 0.001) indicates that correlations between items were high enough for principal component analysis (Table 4).

**TABLE 4:** KMO and Bartlett’s test.

Kaiser–Meyer–Olkin measure of sampling adequacy		0.697
Bartlett’s Test of Sphericity	Approx. Chi-Square	772.72
	Df	91
	Sig	<0.001

Therefore, the construct validity of the Romanian version of the Hill-Bone Compliance to High Blood Pressure Therapy Scale was measured using exploratory factor analysis in SPSS and principal component extraction with promax rotation (because of expected dependency between the components) on all the 14 items of the questionnaire. Factors having an eigen value greater than 1 were extracted. In addition, we used a scree plot to further assess the number of factors.

**FIGURE 1:**
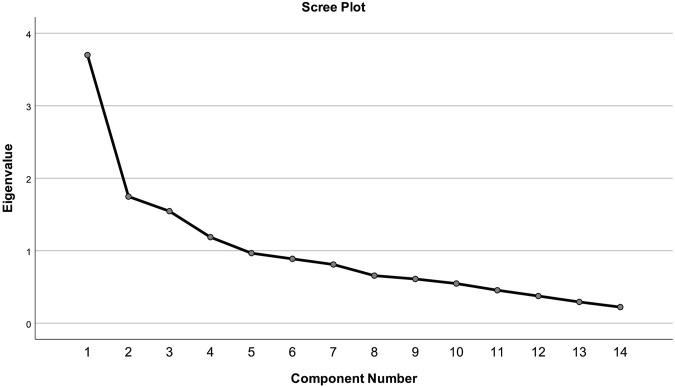
Scree test.

The correlation matrices determinant was 0.020, greater than 0.00001, so none of the variables created any problem. Therefore, all items were considered as correlated and there was no need to remove any items at this stage. Principal component analysis showed a total of four factors having eigen value (sums of squared loadings) greater than one. Similarly, the scree plot test also showed four components with more than 1 eigen value (Figure 1). Therefore, four factors were extracted as per the Kaiser’s criterion. These four factors, in combination, explained 58.4% of the variance. The factor correlation matrix from our analysis showed correlations larger in absolute value than 0.2 (Table 5). Therefore, principal component analysis was conducted on four factors with oblique rotation (promax). Moreover, principal component analysis revealed items retaining in each factor had a loading value greater than 0.4 (Table 6).

**TABLE 5:** Component correlation matrix.

Component	1	2	3	4
1	1	-	-	-
2	.217	1	-	-
3	.274	.209	1	-
4	.254	.364	.277	1

Rotation Method: Promax with Kaiser Normalization.

**TABLE 6:** Exploratory factor analysis by promax rotation with Kaiser’s normalization.

Items	Domain	Component			
		1	2	3	4
1. How often do you forget to take your HBP medicine?	Medicine-taking	-	-	-	0.542
2. How often do you decide NOT to take your HBP medicine?	Medicine-taking	-	0.762	-	-
3. How often do you eat salty food?	Reduce salt intake	-	-	-	0.652
4. How often do you add salt to your food before you eat it?	Reduce salt intake	-	-	-	0.692
5. How often do you eat fast food?	Reduce salt intake	-	-	-	0.506
6. How often do you make the next appointment before you leave the doctor’s office?	Appointment-Keeping	-	-	0.502	-
7. How often do you miss scheduled appointments?	Appointment-Keeping	0.815	-	-	-
8. How often do you forget to get prescriptions filled?	Medicine-taking	0.900	-	-	-
9. How often do you run out of HBP pills?	Medicine-taking	0.679	-	-	-
10. How often do you skip your HBP medicine before you go to the doctor?	Medicine-taking	-	0.648	-	-
11. How often do you miss taking your HBP pills when you feel better?	Medicine-taking	-	0.739	-	-
12. How often do you miss taking your HBP pills when you feel sick?	Medicine-taking	-	0.776	-	-
13. How often do you take someone else’s HBP pills?	Medicine-taking	-	-	0.720	-
14. How often do you miss taking your HBP pills when you are careless?	Medicine-taking	-	-	0.867	-

The original Hill-Bone questionnaire contains three subscales. The distributions of all 14 questions among the four factors are presented in Table 6. Factor 1 retained one appointment keeping and two medicine taking items. However, factor 2 retained four items, all of them from the medicine-taking domain. Factor 3 is similar to factor 1 retaining one item of appointment keeping and two items of the medicine-taking domain of the original tool. In addition, factor 4 retains all three items of reducing sodium or salt intake and one from the medicine taking domain (Table 6).

### 3.7 Known group validity

For this part of our results, firstly we analyzed if overall adherence was significantly associated with the patient having more than one medication per day. Since there is no cut off value in the Hill-Bone Compliance to High Blood Pressure Therapy Scale, we divided the sample into two groups, participants with perfect adherence (score 56) and participants with non-perfect adherence (score <56). Chi-square test was used to measure the correlation between perfect overall adherence and more than one medication user.

Secondly, the same analysis was used comparing the variable more than one medication per day and medicine taking adherence subscale. For the medicine taking adherence subscale a score of 36 was considered perfect adherence, lower than 36 was considered non-perfect adherence. A *p*-value less than 0.05 was considered statistically significant.

Our chi square test in SPSS demonstrated that the overall perfect adherence was not significantly associated with the number of medications taken per day variable (*p* = 0.721). However, a significant association was reported between the medicine taking adherence subscale and the number of medication-taking variable (more than one pill per day) (*p* = 0.004) (Table 7).

**TABLE 7:** Chi-square test of adherence level with medication number.

Medication number	Adherence to medication taking (9 items)		Overall treatment adherence (14 items)	
	Perfect adherence (score = 36)	Non- adherence (score <36)	Perfect adherence (score = 56)	Non- adherence (score <56)
One medication	25	76	2	28
More than one medication	11	101	15	159
Chi square	8.340		0.128	
*p*-value	0.004*		0.721	

*Significant if *p* < 0.05.

## 4 Discussion

For a proper management of hypertension, it is important that patients adhere to the recommendations of healthcare providers regarding lifestyle changes and therapy regimen. Thus, an adapted and validated tool for assessing adherence levels is of much use for professionals.

The purpose of this study was to prepare a Romanian version of the Hill-Bone Compliance to High Blood Pressure Therapy scale and to assess its validity and reliability in a representative group of patients. The instrument was tested in regard to its reliability and validity. No components of the tool were removed, since the reliability assessment highlighted that the internal consistency of the Romanian version of the instrument was 0.733. Other studies of translation and validation of the Hill-Bone Compliance to High Blood Pressure Therapy Scale yielded similar result: the Turkish version obtained a Cronbach alpha coefficient of 0.72 for the 14-item scale (Karademir et al., 2009), the Polish adaptation coefficient was 0.80 (Uchmanowicz et al., 2016), while the Chinese version obtained a coefficient of 0.857 (Pan et al., 2020). Generally, a Cronbach alpha coefficient of internal consistency greater than 0.7 is considered satisfactory and acceptable (Taber, 2018).

Resources which aim at aiding professionals in treatment adherence studies in Romania are scarce and, to our knowledge, this is the first study to translate, adapt and validate the English version of the Hill-Bone Compliance to High Blood Pressure Therapy Scale into Romanian. The statistical analysis, good construct validity and internal consistency highlighted the fact that the tool can measure adherence to treatment among Romanian hypertensive patients, providing thus a useful tool for Romanian health professionals. Although the evaluation was done in a physician’s office, pharmacists could also assess adherence using this scale in community pharmacies, since these are some of the most accessible healthcare locations for the general public in Romania (Negru et al., 2010).

This study has some limitations. It is worth mentioning that although questionnaires are important for assessing adherence as well as for providing additional information in regard to possible reasons for non-adherence, they are indirect measures, which means that they cannot guarantee that patients are indeed taking their medication as recommended. It is also known that by using indirect measures of assessment, patients easily overestimate their adherence levels, while also being subjective to the Hawthorne effect, in which their behaviour changes when they are being observed. This is important since data was collected through interviews. Another aspect which should be noted is the fact that the translation, cultural adaptation and validation process was done using a sample of patients from Northeastern Romania. While the analysis demonstrates the reliability and validity of this instrument for the specific population, its usefulness for all Romanian population groups will need to be determined in further studies.

The Romanian version of the Hill-Bone Compliance to High Blood Pressure Therapy Scale can be used in research as well as in clinical practice. Its ease of use contributes to its possible administration in several healthcare settings. Due to its subscales, it has high clinical utility in personalized care, by developing interventions tailored to patients’ needs. For example, patients that consistently obtain low scores on the salt-intake subscale could benefit from educational interventions on the importance of diet, reducing salt intake or other appropriate lifestyle changes.

Thus, this instrument can be of much use for professionals in evaluating patients’ adherence levels, in proposing ways of obtaining better results in this sense, with the final scope of developing specific interventions aimed at improving patients’ medication taking behavior.

## Data Availability

The raw data supporting the conclusion of this article will be made available by the authors, without undue reservation.
